# The Impact of Coaching on Those Who Coach in Academic Medicine

**DOI:** 10.1007/s40670-025-02416-6

**Published:** 2025-07-04

**Authors:** Jean M. Bailey, Elaine E. Schulte, Wendy L. Ward, Debra Atkisson, Bradley E. Barth, Linda M. Love, Margaret Ann Cary, Nicole M. Deiorio

**Affiliations:** 1 Faculty Development, School of Medicine, Virginia Commonwealth University, Richmond, VA USA; 2 Division of Academic General Pediatrics, Albert Einstein College of Medicine, Bronx, NY USA; 3 Academic Affairs and Faculty Development, The Children’s Hospital at Montefiore, Bronx, NY USA; 4 CHAM Peer Mentoring Program, The Children’s Hospital at Montefiore, Bronx, NY USA; 5 Leadership, Engagement, Advocacy, and Diversity (L.E.A.D.) Program, The Children’s Hospital at Montefiore, Bronx, NY USA; 6 College of Medicine, University of Arkansas for Medical Sciences, Little Rock, AR USA; 7 Academic Affairs, University of Arkansas for Medical Sciences, Little Rock, AR USA; 8 Interprofessional Faculty Development, Academic Affairs, University of Arkansas for Medical Sciences, Little Rock, AR USA; 9 International Coaching Federation, University of Arkansas for Medical Sciences, Little Rock, AR USA; 10 Sr VP Clinical Services, Blended Health, Plano, TX USA; 11 American Psychiatric Association, Washington, DC USA; 12 American Board of Psychiatry & Neurology Psychiatry, Deerfield, USA; 13 Child and Adolescent Psychiatry, American Board of Psychiatry & Neurology, Chicago, USA; 14 International Coaching Federation, Lexington, USA; 15 Medical Center, University of Kansas, Kansas City, KS USA; 16 Faculty Development, University of Nebraska Medical Center, Omaha, NE USA; 17 Family Medicine, Georgetown University of Family Medicine, Washington, DC USA; 18 Emergency Medicine, Student Affairs, Virginia Commonwealth University School of Medicine, Richmond, VA USA

**Keywords:** Coaching, Faculty development, Mentoring

## Abstract

**Introduction:**

As coaching increases in Academic Health Centers (AHCs), a deeper understanding of its benefits is needed. One gap is understanding potential impact on coaches themselves. As coaching is grounded in principles of Appreciative Inquiry (positivity begets positivity), coaching may also benefit coaches. Answering this question will allow for broader assessment of return on investment of coaching programs.

**Methods:**

The authors developed an electronic survey using the theoretical framework of appreciative inquiry, collecting demographics and elements of the burnout scale rating (BSR), value of work rating (VWR), job satisfaction rating (JSR), and free-text benefits of being a coach. Using convenience sampling, the survey was distributed to coaches of physicians through author affiliations with coaching groups and listservs from the Association of American Medical Colleges (AAMC) Group on Faculty Affairs (GFA), an international coaching organization, and an AHC coaching group. Quantitative data were descriptively analyzed. Free-text answers underwent inductive thematic analysis.

**Results:**

Eighty-nine of 433 survey viewers (21%) completed it. The majority feel valued, experience minimal feelings of burnout, and endorse job satisfaction. Analysis identified three major benefits: personal fulfillment, interpersonal benefit, and professional growth and advancement.

**Discussion:**

Coaching positively impacts coaches themselves, adding evidence for its use in AHCs. Many coaches find their work deeply fulfilling as it aligns with their values, enhances self-awareness, and improves their wellbeing by fostering mindfulness and personal growth. Additionally, coaching strengthens their communication and leadership skills leading to better interactions with patients and trainees, professional development, and more effective leadership.

## Introduction

Coaching is a popular professional development tool that supports people to achieve goals and improve job performance. Coaching aims to benefit any person by “inspiring them…to maximize their personal and professional potential” [1]. This is done through partnering a coach with a coaching client and exploring topics and areas of interest using thought-provoking questions to tap into the coaching client’s imagination, productivity, and leadership [2]. Because coaching serves to support any desire for change the coaching client brings to the relationship, coaching in academic medicine can be harnessed for creating actionable change across all levels of current and future physicians in such domains as professional identity formation, clinical skill development, communication skills, leadership skills, career transitions, and wellbeing [3, 4]. As coaching in academic medicine continues to grow, it is necessary to more fully understand the benefits of the coaching relationship and its returns on investment.

Coaching physicians can come with its own set of challenges, and there are additional competencies necessary to competently coach those on the physician continuum [5]. However, given that coaching is grounded in the positive psychology framework of Appreciative Inquiry, which supports that positive change comes from positive actions [6, 7], it is reasonable to hypothesize that the act of providing coaching would bring positive change not just in the coachee, but in the coach as well. Earlier evidence supports this hypothesis in the domain of coaching in medical education [8, 9]. As coaching has exploded within medical education in the past 5 years, additional evidence has mounted about the potential benefits of coaching for the coaches [8, 10, 11]. Brooks et al. found through a qualitative study of coaches for medical students at a single school that coaches endorsed a personal benefit of “connectivity” [10]. In a mixed-methods study, Elster et al. studied undergraduate medical education (UME) coaches at a single school who reported coaches had higher self-efficacy in some educational domains when compared to those not coaching; there was some relationship to job satisfaction; however, two-thirds of coaches reported burnout, perhaps related to identity challenges in this role [11]. Coaches in a pediatric residency noted a positive effect on their own professional identity formation as found in a qualitative study by Selling et al. [8] A more recent qualitative study across three UME programs described the positive and negative effects of coaching on the coach when supporting struggling learners [12]. However, these studies are limited to single sites and/or specific coachee segments.

There are a number of types and styles of coaching as well as coaching methodologies and frameworks. Although not licensed by any federal or state regulatory body, many professional coaches may or may not have undergone specific educational training with certain experiential requirements [13]. As such, coaches have a wide variety of approaches and practices. Regardless of their title(s), coaches who coach in AHCs come from different experiential backgrounds and may apply coaching skills in a variety of ways (e.g. academic coaching, residency program coaching). It is unlikely that all coaches “coach” the same way; however, they are generally motivated to support others in their personal and professional growth.

This present study aims to expand upon the existing literature by sampling coaches across multiple sites throughout the USA who coach multiple different types of people in academic medicine, including learners, faculty, and other healthcare professionals. Understanding potential benefits to coaches will support leaders in understanding the value that implementing coaching programs may add to their academic healthcare environment and assist in the recruitment of coaches for academic coaching programs. Using a conceptual framework of Appreciative Inquiry, we strove to answer the question: what benefits to themselves do coaches report from coaching clients in academic medical centers? We hypothesize that coaches will report multiple types of individual benefits from their coaching practice, including personal professional development, career success, and professional and personal wellbeing.

## Methods

### Overview

In 2023–2024, we performed a mixed-methods survey study. A mixed-methods study in qualitative research is a research approach that combines both quantitative (numbers, statistics) and qualitative (experiences, opinions, motivations) methods within the same study to answer a research question more thoroughly than using just one method alone [14]. The research team is a group of eight faculty who are certified and/or credentialed coaches working in academic health (AHC) settings.

### Survey Instrument

The research team developed an 18-question mixed-methods online survey using QuestionPro electronic data capture tool to gather demographic data and perceptions of coaching impact on their own patient care, teaching and learning, research, leadership, career advancement, and wellness. There were also questions regarding perceived benefits of being a coach, and elements from a burnout scale rating (BSR) [15], a value of work rating (VWR) [16], and a job satisfaction rating (JSR) [17]. The survey was developed iteratively and attained agreement in our expert author group. Pilot testing was not performed. Participants responded to the survey using multiple choice responses, Likert scale ratings, and narrative responses. The survey is available as Appendix.

### Sampling Strategy and Participants

A convenience sample of potential respondents was identified through team member affiliations with coaching organizations, AHC coaching programs, institutional coaching programs, and other professional groups. Participants were also recruited using listservs from the Association of American Medical Colleges (AAMC) Group on Faculty Affairs (GFA), an international coaching organization, and an Academic Health Center (AHC) coaching group. There is no national registry of coaches, much less a registry of those working in academic medical centers, so based on the study group expertise we speculate an estimated 300–500 coaches are working in AHCs.

Research team members sent an email describing the study with a link to the survey to the above groups in December 2023. Potential participants were told the survey would likely take no longer than 20 min to complete. Self-selecting respondents had a 2-week window to respond, and a reminder email from the project representatives was sent after the first week.

### Data Analysis

Data was exported from QuestionPro into an Excel spreadsheet (Microsoft Corporation, 2018.) Quantitative data regarding demographics, BSR, VWR, and JSR were descriptively analyzed.

Inductive analysis was performed on the free-text data. We chose to apply inductive thematic analysis as it can be used to “understand experiences, thoughts or behaviors across a data set” [18] by revealing and analyzing themes to produce results; the process is data driven so themes are strongly linked to the data [19] rather than being manipulated to neatly map back to the study’s conceptual framework or research questions. For our study, each survey question with correlating responses were assigned to a subset of two team researchers to identify initial codes. A second review of codes was conducted by a different set of researchers to begin to categorize codes and identify potential themes. A final analysis was conducted by team leaders to identify themes from the categories showing the impact of the coaching work on the coaches. Lastly, Google Notebook LM (Google, 2024) was used to generate themes for comparison to the initial themes identified by the researchers. Team members determined that thematic sufficiency was reached; Google LM was also applied to check for sufficiency and agreed with the team’s determination. Team members chose representative quotations for each theme.

### Reflexivity

Each step of the project (methods planning, survey development, coding, and manuscript preparation) underwent multiple iterations to ensure all perspectives within the team were captured. Our team is composed of faculty in academic medical centers who all hold training and certification credentialing as coaches.

### Research Ethics Statement

The study received IRB approval from an Institutional Review Board.

## Results

Of the 433 people who viewed the survey, 89 respondents (21%) completed the survey; 3 additional respondents partially completed the survey. Respondent demographics are shown in Table 1.

**Table 1:** Study participant demographics and coaching experience

Survey distribution	Viewed	Responses	Completed	Ave. time to complete			
*n* (%)	433	92	89 (97%)	7 min			
Years as coach	< 1	1–4.9	5–10	> 10			
*n* (%)	9 (10%)	48 (53%)	22 (24%)	12 (13%)			
Hours of coach training	None	< 30 h	31–60 h	61–100 h	> 100 h		
*n* (%)	17 (19%)	15 (17%)	12 (13%)	15 (17%)	30 (34%)		
Whom do you coach	Colleagues	Executives	Healthcare professionals	Medical students	Trainees	Other-1	
*n* (%) (Check all that apply; percentages add up to > 100)	45 (51%)	23 (%)	55 (25%)	52 (24%)	29 (13%)	12 (5%)	
What is your role in AMC	Administrator	Clinician	Coach	Educator	Physician	Researcher	Other—2
*n* (%) (Check all that apply; percentages may add up to > 100)	35 (39%)	37 (42%)	49 (55%)	49 (55%)	52 (58%)	23 (26%)	10 (11%)
What is your age?	25–29	40–54	55–69	70 +			
*n* (%)	9 (10%)	42 (47%)	32 (36%)	6 (7%)			
What is your gender identity	Female	Male	Non-binary	Other/Prefer not to say			
*n* (%)	64 (72%)	25 (28%)	0	0			

Other-1: Community Professionals; Faculty & Faculty Leaders; Physician Leaders & Academic Faculty; Non-Healthcare Professionals; Medical School Support Staff; Healthcare Information Technology Professionals; Advanced Practice Providers; Nurse Leaders; Associate Deans; Administrative Employees

Other—2: External Coach; Team Development Facilitator; Pharmacist; Chief Wellness Officer for College of Medicine; Retired; Senior Associate Dean; Independent Contractor; Self Employed

### Data from Wellness Instruments

Table 2 depicts the results from the BSR, VWR, and JSR items. The majority of respondents indicated that they feel valued, experience minimal feelings of burnout, and are satisfied with their jobs.

**Table 2:** Wellness instrument results (*NIH*, National Institutes of Health; *NIOSH*, National Institute for Occupational Safety and Health)

Burnout Scale Rating (Dolan, 2014)	Number agreeing with statement (percent)
I enjoy my work; I have no symptoms of burnout	25 (41.67)
Occasionally I am under stress and I don’t always have as much energy as I once did, but I don’t feel burned out	25 (41.67)
I am definitely burning out and have one or more symptoms of burnout such as physical and emotional exhaustion	9 (15)
The symptoms of burnout that I’m experiencing won’t go away; I am frustrated at work a lot	1 (1.67)
I feel completely burned out and often wonder if I can go on; I am at the point of where I may need some changes or may need to seek some sort of help	0 (0)
**Value of Work Rating (NIH 2000)**	
**How often do you feel that what you do is valuable and worthwhile?**	*N* (percent)
Never	0 (0)
Once in a while	1 (1.67)
About half the time	5 (8.33)
Most of the time	38 (63.33)
Always	16 (26.67)
**Quality of Worklife Module (NIOSH, 2021)**	
**How satisfied would you say you are with your job?**	N (percent)
Not at all satisfied	0 (0)
Not too satisfied	2 (3.33)
Somewhat satisfied	16 (26.67)
Very satisfied	42 (70)

### Qualitative Data: Major Themes

The qualitative survey data revealed the three major themes related to the benefits of coaching for the coach: personal benefit, interpersonal benefit, and benefit to professional growth and advancement. Representative quotes are shown in Table 3.

**Table 3:** Selected qualitative quotations by theme

Personal value	Interpersonal benefit	Professional growth and advancement
As I have learned more about being a coach, I have listened better to those around me, including myself, and have toned down my inner self critic	... coaching has me more intentionally setting expectations and goals with all the learners I work with in the clinical (and non-clinical) setting and how to best ask questions not only during these sessions but in every day interactions on to get them to think about how they best learn and accomplish their goals. I also think about how to best loop back for feedback and coaching for next steps	Fulfilling–my coach training prompted a change in career path towards certification as a chief wellness officer, and I have subsequently been appointed the Chief Physician Wellness Officer for my institution. g career pivot
It’s hard to coach others toward resilience, balance and wellness and not have one’s own practices and being impacted	The skills of non-judgmental active inquiry have directly impacted my work in the emergency department. I am more open and a better listener following my coaching training	It has accelerated my leadership career. Coaching skills both make me a better leader, but also a better listener and I can help people understand and identify their situation/desire/needs/goals
In the face of widespread career dissatisfaction, I am happier than ever, in no small part due to my ability to self-coach and be coached. And I am so happy with my new-ish role as a wellbeing leader and feel like the work is extremely important and innovative, which has added so much purpose in this last decade of my career	A coach-approach serves in leading a team, managing meetings, and developing my proteges. It also allows for a flatter organizational hierarchy which has improved feedback and created better understanding of the most important issues and impacts of policy and change	Life-saver for me as full retirement would be impossible. I thrive on the engagement with students and faculty but at a pace I can manage at 75 yrs
Being able to help others find their purpose or meaning in their work and lives is my purpose. I have been able to fulfill my purpose and increase my impact through coaching and coach education	It has helped me understand people, personalities and approaches to better interact with a wider variety of individuals	Immensely! Deep listening & empowering others; not needing to be the one who has the answers but coaching others to find the answers within themselves. Through my leadership role within an academic health center’s research institute, the support of coaching and other serendipitous events, I shifted my career to becoming a coach full-time (over a decade ago) to others within healthcare
As I have gone through the coaching training I have been able to reassess my strengths and weaknesses. This has made me more comfortable stretching myself	I really try to meet people where they’re at as opposed to where I want them to be	Able to publish some coaching research which helped promote and expand my network

#### Personal Fulfillment

Many coaches find their coaching work rewarding with some describing it as a “fulfilling career pivot.” They reported receiving positive feedback about their coaching, and they appreciate being able to guide others and witness the growth of coachees. Some coaches noted that coaching aligns with their values and gives them a sense of purpose leading to increased job satisfaction. Coaches also stated that they felt a level of enhanced self-awareness and personal growth and shared that engaging in coaching prompts introspection and self-reflection. They shared that this heightened self-awareness led to a better understanding of their own beliefs and actions. Some coaches reported becoming more mindful and better listeners in their personal lives due to coaching.

Coaching also impacts stress and wellbeing. Many coaches found that coaching contributed positively in their wellbeing. They shared how they apply coaching principles to support their own mental health and experience the process of coaching as almost a “detachment” or “meditative” state that helps them to engage with others in a different, more focused way.

Some coaches attributed their increased job satisfaction, purpose, and skills to coaching. Not only does coaching offer them a new focus and variety to their work, but it also provides them with a renewed sense of curiosity, interest, and engagement and boosts their feelings of fulfillment.

#### Interpersonal Benefit–Improved Interactions

Responses showed that coaches are able to communicate better by asking better questions and listening better, and they realize that it is not necessary to have solutions. This helps them focus on listening which results in their being a better partner with patients, enhances their curiosity, and helps them listen to the needs of their team.

Coaches reported positive impacts on their patient interactions, primarily in terms of improved communication and a more patient-centered approach. Some respondents mentioned that coaching helped them become better listeners, ask more open-ended questions, and be more validating of patients’ experiences. Others highlighted that coaching provided a framework for understanding patients’ perspectives, needs, and goals, ultimately enhancing the patient experience.

Coaches involved in medical education described using coaching skills to enhance their interactions with trainees (students, residents, and fellows). They noted a shift from providing direct answers to facilitating problem-solving and critical thinking in trainees. Coaching helped them ask better questions, guide trainees in setting goals, and tailor their teaching to individual trainee needs. They found they practice more curious listening, are more observant, and are more encouraging and empowering with their trainees.

#### Professional Growth and Advancement

Coaching others often translates into improved skills and insights gained through the coaching process that coaches can apply to their own careers, which contributed to their professional development and even promotions. Coaches reported benefitting from research and publishing opportunities helping them to make connections with a new focus, further enhancing their careers.

Coaches in leadership roles emphasized the positive impact of coaching on their leadership styles and effectiveness. They reported improved listening skills, increased empathy, and a greater ability to empower and support others. Some specifically noted that coaching helped them navigate difficult conversations, manage conflict, and provide effective feedback. It also helped them develop new approaches to problems as leaders, by listening to how other leaders approach similar issues.

## Discussion

Coaching, as a practice to support medical trainees and healthcare professionals, continues to grow in popularity in academic medicine [13, 20]. Our findings offer preliminary evidence that coaches within AMCs find benefit from the practice to themselves. This study explored the unique perspectives of coaches who are coaching in academic medicine today. While coaching aims to benefit the coachee [3, 13], this study clearly delineates that the coaches also derive multiple benefits from their coaching practice and adds to the emerging literature in this regard [11, 21].

Other studies have found enhanced self-efficacy and job satisfaction among coaches [11]. In our present study, the benefits found were much broader. Coaches described themselves as being more reflective, cognitively nimble, relationship-centered, and fulfilled by coaching others. Coaches recognized the duality in benefit, meaning that as they coach and develop others, they themselves grow and develop. In particular, respondents saw enhancements in communication skills, leadership skills, a deep sense of purpose, and meaningful connections to both patients and trainees. In addition, the impact of coaching had a positive impact on their self-perceived value as well as other-perceived values such as in promotion. Unlike other studies who found burnout rates remain high for clinician coaches [11, 21], this study found coaches had an improved sense of wellbeing and a mitigation of their stress from the experience of coaching others. The enhanced understanding of the impact of coaching might be related to the qualitative approach and use of appreciative inquiry to develop and implement the present survey [22] and the broad sample of coaches who work with both faculty and trainees rather than the more narrow samples of women surgeons [21] and educators [11].

While this study expands our understanding of the impact of coaching on coaches and their medical institutions, there are some limitations worthy of note. The validity of our survey tool may be different from the original tools as we selected only certain items from the VWR and JSR instruments. The study is centered on the self-report of coaches currently practicing in AHCs. This study may have a sampling bias in that it does not account for perspectives of coaches who may have left coaching or who chose not to respond to the survey, which may offer additional information. For example, coaches who are suffering from burnout may have been less likely to participate in our study. No evidence of burnout in coaches was noted in this survey, but it would be prudent to monitor as coaching expands in AHCs. Our sample group is heterogenous, such as in whom they coach and how they have been trained. However, coaching principles and competencies are not related to these demographics, our findings should still have relevance. Thematic sufficiency was reached for the qualitative arm of the study. This study highlights the rewards and advantages coaches experience in their work as coaches and offers an early look at the impact on coaches; however, additional study may be needed regarding the sustainability of these positive results as well as comparative impact across different coach characteristics (different subspecialties, different institutional settings, researchers, educators, clinicians, etc.). Additionally, a coach’s own coaching training, type of coaching provided (process/facilitation-based or outcome/goal-based ^(22)^), and background (personal identities such as gender, race, culture, etc.) may influence their experiences and perspectives. We are unsure how much time the respondents spent on coaching. Investigation of the differential impacts of number of sessions, duration of coaching, and format of coaching (face-to-face coaching vs virtual coaching; individual vs group coaching) in medical settings is also needed. It would be an interesting area of future study to understand which subgroups of coaches found which benefits. Coaching practices vary widely across organizations, and structural, compensation, workload, coaching themes, or other variables may impact coach experiences over time warranting further study. For example, their descriptions of personal advantages in their work as coaches uniformly suggest that coaching applications within AHCs would add a return on investment to coaches in addition to coachees.

## Conclusion

This mixed-methods survey study offers additional evidence of the positive impact of coaching in academic health centers, demonstrating the value of coaching for the coaches themselves. Organizational leaders tasked with implementing or maintaining coaching programs can use these findings to consider the additional benefits to coaches when determining the value of a coaching program.

## Data Availability

Survey data is available by contacting the corresponding author.

## Appendix 1. Survey Questions

### Figure 1. Survey Questions

1. How long have you been a coach?
  1. Less than 1 year
  2. 1 to 5 years
  3. 5 to 10 years
  4. More than 10 years
2. How many hours of coaching certification training have you completed?
  1. No certified training
  2. 1 to 30 hours
  3. 31 to 60 hours
  4. 61 to 100 hours
  5. More than 100 hours
3. Where do you coach? (Check all that apply)
  1. Inside the organization/institution
  2. Outside the organization/institution
  3. Both inside and outside the organization/institution
  4. I am not currently coaching
4. Whom do you coach? (Check all that apply)
  1. Colleagues
  2. Executives
  3. Healthcare professionals
  4. Medical students
  5. Trainees (graduate students, residents, fellows)
  6. Other
5. What is your role at your academic health center? (Check all that apply)
  1. Administrator
  2. Clinician
  3. Coach
  4. Educator
  5. Physician
  6. Researcher
  7. Other
6. What is your age?
  1. 25 to 39
  2. 40 to 54
  3. 55 to 69
  4. 70+
7. What is your gender identity?
  1. Female
  2. Male
  3. Non-binary
  4. Prefer not to say

How have the experience and skills you've gained from coaching impacted your experiences and approaches in the following areas? (Respond to each that applies)

8. Patient Care

9. Teaching and Learning

10. Leadership

11. Research

How has the practice of coaching influenced you in the following ways? (Respond to each that applies)

12. Career Satisfaction

13. Career Advancement/Promotion

14. Wellness

15. What do you perceive as the most important/direct benefit(s) of your being a coach?

16. Based on your definition of burnout, how would you rate yourself? (Dolan et al. 2014)
  1. I enjoy my work; I have no symptoms of burnout
  2. Occasionally I am under stress and I don’t always have as much energy as I once did, but I don’t feel burned out
  3. I am definitely burning out and have one or more symptoms of burnout such as physical and emotional exhaustion
  4. The symptoms of burnout that I’m experiencing won’t go away; I am frustrated at work a lot
  5. I feel completely burned out and often wonder if I can go on; I am at the point of where I may need some changes or may need to seek some sort of help
17. How often do you feel that what you do is valuable and worthwhile? (NIH Toolbox)
  1. Never
  2. Once in a while
  3. About half the time
  4. Most of the time
  5. Always
18. How satisfied would you say you are with your job? (Quality of Worklife Module NIOSH 2010)
  1. Not at all satisfied
  2. Not too satisfied
  3. Somewhat satisfied
  4. Very satisfied
